# The role of diffusion and perfusion weighted imaging in the differential diagnosis of cerebral tumors: a review and future perspectives

**DOI:** 10.1186/1470-7330-14-20

**Published:** 2014-04-29

**Authors:** Patricia Svolos, Evanthia Kousi, Eftychia Kapsalaki, Kyriaki Theodorou, Ioannis Fezoulidis, Constantin Kappas, Ioannis Tsougos

**Affiliations:** 1 Medical Physics Department, University of Thessaly, Biopolis, 41110 Larissa, Greece; 2 Department of Radiology, University Hospital of Larissa, Biopolis, 41110 Larissa, Greece

**Keywords:** Diffusion weighted imaging, Diffusion tensor imaging, Dynamic-susceptibility contrast imaging, Brain tumors, Differential diagnosis

## Abstract

The role of conventional Magnetic Resonance Imaging (MRI) in the detection of cerebral tumors has been well established. However its excellent soft tissue visualization and variety of imaging sequences are in many cases non-specific for the assessment of brain tumor grading. Hence, advanced MRI techniques, like Diffusion-Weighted Imaging (DWI), Diffusion Tensor Imaging (DTI) and Dynamic-Susceptibility Contrast Imaging (DSCI), which are based on different contrast principles, have been used in the clinical routine to improve diagnostic accuracy. The variety of quantitative information derived from these techniques provides significant structural and functional information in a cellular level, highlighting aspects of the underlying brain pathophysiology. The present work, reviews physical principles and recent results obtained using DWI/DTI and DSCI, in tumor characterization and grading of the most common cerebral neoplasms, and discusses how the available MR quantitative data can be utilized through advanced methods of analysis, in order to optimize clinical decision making.

## Introduction

Magnetic Resonance Imaging (MRI) has evolved to the most important non-invasive diagnostic tool for the detection, presurgical planning and evaluation of treatment response of cerebral tumors. Despite its excellent soft tissue visualization and variety of imaging sequences, conventional MRI presents limitations regarding certain tumor properties, such as infiltration and grading [1]. The inability to detect infiltrating cells beyond the tumoral margin and to accurately define the grade of the tumor impedes surgical resection and the post-surgical treatment procedure. Hence, biopsy remains the gold standard, although it might provide histo-pathological information about a limited portion of the lesion and not necessarily about the whole neoplastic tissue. Therefore, advanced MRI techniques using different contrast principles, have been incorporated into the clinical routine in order to aid tumor diagnosis. Diffusion-Weighted Imaging (DWI), Diffusion Tensor Imaging (DTI) and Dynamic-Susceptibility Contrast Imaging (DSCI) provide non-invasively significant structural and functional information in a cellular level, highlighting aspects of the underlying brain patho-physiology.

The possibility to characterize tumoral and peritumoral tissue microstructure, based on water diffusion and perfusion findings, provided clinicians a whole new perspective on improving the management of brain tumors. A large number of studies have been conducted in order to assess whether DWI, DTI, and DSCI and the quantitative information derived by these techniques, aid differential diagnosis, especially in cases of ambiguous cerebral neoplasms. Many researchers have reported increased diagnostic value when using DWI and/or DTI, and/or DSCI for tumor differentiation [2-6]; however the number of studies reporting otherwise remains significant [7-11]. The most probable explanation may be the complexity of the underlying pathophysiology, resulting in similar diffusion and perfusion patterns, and thus to controversial observations. The present work, reviews recent results that have been obtained using DWI/DTI and DSCI, in tumor characterization and grading of the most common cerebral neoplasms, and discusses how the available quantitative data-information can be exploited through advanced methods of analysis, in order to optimize clinical decision-making.

## Review

### Diffusion and perfusion imaging: basic principles

#### Diffusion weighted imaging

The random motion of water molecules inside a medium, due to their thermal energy, is described by the “Brownian” law. Diffusion is considered the result of the random movement of water molecules [12]. Diffusion occurs at equal rates in all directions inside an isotropic medium, however within tissues water motion is restricted. Therefore, inside a complex environment, such as the human brain, cell membranes, neuronal axons and other macromolecules, act as biological barriers to free water motion, hence water mobility is considered to be anisotropic. Specifically, the highly organized white-matter bundles, due to their myelin sheaths, force water to move along their axes, rather than perpendicular to them.

Diffusion Weighted Imaging is an advanced MR imaging technique, which uses the Brownian motion of molecules to acquire images. When the patient is inserted into the magnet bore, the nuclear spins are lined up along the direction of the static magnetic field. If a radiofrequency pulse is applied, the protons will spin at different rates depending on the strength, duration and direction of the gradient. If an equal and opposite gradient is applied the protons will be refocused. Stationary protons will provide a null signal after this counter-process. On the contrary, mobile protons that have changed position during the time period between the two gradients, will present a signal loss, that is dependent on the degree of diffusion weighting, referred to as the b-value [13]. Therefore, measuring the signal of the mobile protons allows determination of the amount of diffusion, which has occurred in a specific direction. The b-value is described by the following mathematical equation:

(1) $$b={\left(\mathrm{\gamma G\delta}\right)}^{2}\left(\Delta -\frac{\delta }{3}\right)$$

Δ is the temporal separation of the gradient pulses, *δ* is their duration, *G* is the gradient amplitude and *γ* is the gyromagnetic ratio of protons [14]. The diffusion time is assigned as (Δ-δ/3), where the second term in the expression accounts for the finite duration of the pulsed field gradients. The units for the b-value are smm^-2^, and the range of values typically used in clinical diffusion weighting is 800–1500 smm^-2^. This range of b-values is considered reasonable based on contrast-to-noise ratio (CNR) estimates at gradient strengths of clinical MR instruments [15].

For a fixed diffusion weighting it can be shown that the signal in a diffusion-weighted experiment is given by the equation:

(2) $$S=S_{0}e^{-\mathrm{TE}/T2}e^{-\mathrm{bD}}$$

Sο is the signal intensity in the absence of any T2 or diffusion weighting, TE is the echo time and D is the apparent diffusivity, either called the Apparent Diffusion Coefficient (ADC). It is called “apparent” because it is often an average measure of much more complicated processes inside the tissues, and does not reflect the magnitude of intrinsic self-diffusivity of water per se [16,17]. The first exponential term in Equation 2 is the weighting due to transverse (T2) relaxation and the second term shows that diffusion induces an exponential attenuation to the signal [12]. As the diffusing spins are moving inside the field gradient, the field affects each spin differently, thus the alignment of the spins with each other is destroyed. Since the measured signal is a summation of tiny signals from all individual spins, the misalignment, or “dephasing”, caused by the gradient pulses results in a drop in signal intensity; the longer the diffusion distance, the lower the signal (more dephasing) [15].

The magnitude of diffusion within each voxel can be measured by the ADC. A parametric map of ADC values can be obtained by collecting a series of DW images with different b-values. The intensity of each image pixel on the ADC map reflects the strength of diffusion in the pixel. Therefore, a low value of ADC (dark signal) indicates that water movement is restricted, whereas a high value (bright signal) of ADC represents free diffusion in the sampled tissue [18]. For example, in cerebral regions where water diffuses freely, such as CSF inside the ventricles, there is a drop in signal on the acquired DW images, whereas in areas that contain many more cellular structures and constituents (grey matter or white matter), water motion is relatively restricted and the signal on DW images is increased. Consequently, regions of CSF will present higher ADC values on the parametric maps, than other brain tissues.

Single-shot echo-planar imaging (EPI) is the most widely used diffusion-weighted acquisition technique. It is fast and insensitive to small motion, and readily available on most clinical MRI scanners. However, EPI is sensitive to magnetic field inhomogeneities, which cause distortions in the image data. Alternative diffusion-weighted imaging techniques include multi-shot EPI with navigator echo correction or diffusion-weighted PROPELLER and parallel imaging methods, such as SENSE [19]. The application of such techniques increase the bandwidth per voxel in the phase encode direction, thus reducing artifacts arising from field inhomogeneities, like those induced by eddy currents and local susceptibility gradients.

DWI has been considered useful, however there is a limitation that should be taken into account. The DWI sequence is sensitive, but not specific for the detection of restricted diffusion, and one should not use only signal changes to quantify diffusion properties, as the signal from DWI is prone to the underlying T2-weighted signal, referred to as the “T2 shine-through” effect. Specifically, on T2-weighted images the increased signal in areas of cytotoxic edema may be present on the DWI images as well [18]. To determine if this signal hyperintensity on DWI images truly represents decreased diffusion, an ADC map should be used. The ADC sequence is not as sensitive as the DWI sequence for restricted diffusion, but it is more specific, as the ADC images are not susceptible to the “T2 shine-through” effect [18].

### Diffusion tensor imaging

In DWI water diffusion is considered an isotropic process. However, as mentioned previously, this is not the case regarding diffusion in the brain, which has natural intracellular (neurofilaments and organelles) and extracellular (glial cells and myelin sheaths) barriers that impede diffusion towards any direction. Water molecules diffuse mainly along the direction of white matter axons, rather than perpendicular to them. Under these circumstances, diffusion becomes highly directional along the length of the tract, and is called anisotropic [12].

Diffusion Tensor Imaging represents a further development of DWI, taking advantage of this preferential water diffusion inside the brain tissue [20,21]. DTI measures both the magnitude and the direction of proton movement within the voxel for multiple dimensions of movement, using a mathematical model to represent this information, called the diffusion tensor [18]. Hence, the directional movement of water molecules inside a voxel can be represented by an ellipsoid, which in turns can be described by the tensor in that specific voxel. The tensor consists of a 3 × 3 matrix derived from diffusivity measurements in at least six different directions. The tensor matrix is diagonally symmetric (D_ij_ = D_ji_) meaning that the matrix is fully determined by six parameters. If the tensor is completely aligned with the anisotropic medium then the off-diagonal elements become zero and the tensor is diagonalized. This diagonilization provides three eigenvectors that describe the orientation of the three axes of the ellipsoid, and three eigenvalues that represent the magnitude of the axes (apparent diffusivities) in the corresponding directions. The major axis is considered to be oriented in the direction of maximum diffusivity, which has been shown to coincide with tract orientation [13,19]. Therefore, there is a transition through the diffusion tensor from the x, y, z coordinate system defined by the scanner’s geometry, to a new independent coordinate system, in which axes are dictated by the directional diffusivity information. Depending on the local diffusion the ellipsoid may be “prolate”, “oblate” or “spherical”. Prolate shapes are expected in highly organized tracts where the fiber bundles all have similar orientations, oblate shapes are expected when fiber orientations are more variable but remain limited to a single plane, whereas spherical shapes are expected in areas that allow isotropic diffusion [22].

The quantification of the local diffusion anisotropy is reflected though the calculation of ‘rotationally invariant’ parameters. Although there are several indices that can be derived from DTI, the most commonly reported are Mean Diffusivity (MD) and Fractional Anisotropy (FA). MD is the mean of the eigenvalues, and represents a directionally measured average of water diffusivity, whereas FA derives from the standard deviation of the three eigenvalues. The signal brightness of a voxel on an FA map, describes the degree of anisotropy in the given voxel. FA ranges from 0 to 1, depending on the underlying tissue architecture. A value closer to 0 indicates that the diffusion in the voxel is isotropic (unrestricted water movement), such as in areas of CSF, whereas a value closer to 1 describes a highly anisotropic medium, where water molecules diffuse along a single axis, such as in the corpus callosum [12].

A further representation of diffusion directionality in various regions of interest is given by the Directionally Encoded Colour (DEC) FA maps. Specifically, the orientation of the ellipsoid in each voxel, defined by the eigenvector with the largest eigenvalue, can be colour-coded to communicate and display information about the direction of white matter tracts. Hence, ellipsoids describing diffusion from left to right are coloured red (x-axis), ellipsoids describing anterioposterior (y-axis) diffusion are coloured green, and diffusion in the craniocaudal direction is coloured blue (z-axis) [23]. This procedure provides a convenient summary map from which the degree of anisotropy (signal brightness) and the fiber orientation in the voxel (colour hue) may be determined. An experienced user can combine and correlate this information with normal brain anatomy, identify specific white matter tracts and assess the impact of a lesion on neighbouring white matter fibers.

As previously mentioned, diffusion anisotropy is dictated by the underlying tissue structure, and mainly from the white matter architecture. This correlation enables the mapping of white matter tracts non-invasively [24]. Following the tensor’s orientation on a voxel-by-voxel basis, it is possible to identify intravoxel connections and display specific fiber tracts using computer graphic techniques. This process is referred to as DT Tractography. A variety of tractography techniques have been reported [25-28]. All these techniques use mathematical models to identify neighbouring voxels, which might be located within the same fiber tract based on the regional tensor orientations and relative positions of the voxels. Towards this direction a number of studies have created atlases of the human brain based on DTI and tractography [29,30]. Hence, the displacement or disruption of a specific fiber tract by a tumor may be assessed by 3D tractograms, providing useful information in terms of pre-surgical planning [31,32]. Nonetheless, these techniques present limitations such as in cases of complex tracts (crossing or branching fibers), which should be taken into consideration when these methods are used for preoperative guidance.

Unlike DWI, the diffusion gradients in DTI are applied in multiple directions. Based on previous reports, the number of non-collinear gradients applied varies (ranging from 6 to 55) however an optimal number has not been defined [33-35]. The main drawback, however, of an increased number of gradients in DTI is the imaging time, which increases simultaneously, and may not be useful in clinical practice [36]. Therefore, a trade-off between the imaging time and the number of gradients applied, in order to obtain sufficient diffusion information, should be established.

### Dynamic-susceptibility contrast imaging

Perfusion refers to the capillary blood supply of a tissue and perfusion MRI enables the measurement of this microcirculation. Dynamic susceptibility contrast imaging (DSCI) is one of the most commonly used techniques for perfusion quantification. DSCI utilizes very rapid imaging to capture the first pass of intravenously injected paramagnetic contrast agent. In DSCI a volume of tissue is imaged repeatedly using an EPI sequence. After a few images have been collected as a baseline, a bolus of gadolinium (Gd)-based chelate is injected as fast as possible. During the first pass through the intracranial circulation, Gd remains in the vasculature and due to its paramagnetic properties causes a reduction of T2 and T2*, which is seen as a dramatic drop in signal intensity on T2-weighted or T2*-weighted mages [37]. The second pass may also be detected as a slight drop in intensity, before the signal returns to baseline. Gradient Echo (GE) and Spin Echo (SE) EPI sequences are used in first-pass perfusion-weighted MRI. The SE is sensitive to detect tumor vascularity at the capillary level. Whereas GE is sensitive to the total blood volume contained in both capillaries and large vessels [1,38]. Tumoral lesions such as gliomas, meningiomas and lymphomas contain both types of vessels therefore GE-EPI technique is more suitable to assess tumor vascularity.

In DSC images the drop in signal is proportional to the concentration of contrast agent and the tissue vascularity. The contrast agent concentration is proportional to the change in relaxation rate ΔR2* (i.e. the change in the reciprocal of T2*), which is can be calculated by the following equation:

$$\mathrm{\Delta R}2^{*}=\frac{-\ln[S\left(t\right)/S_{0}}{\mathrm{TE}}$$

Where S(t) is the pixel signal intensity at time t, S_0_ is the pre-contrast signal and TE is the echo time. This equation is only valid if T1 enhancement associated with Blood-Brain Barrier (BBB) disruption has a negligible effect on signal intensity, which is ensured by using either long TRs, low flip angles, or a combination of the two to reduce saturation [39]. However, this assumption is violated in the case of tumor incidence. These effects can be reduced by fitting a gamma-variate function to the measured ΔR2* curve. The gamma-variate function approximates the curve that would have been obtained without recirculation or leakage [39].

The hemodynamic parameters derived from dynamic MR images are usually the Cerebral Blood Volume (CBV), Cerebral Blood Flow (CBF) and Mean Transit Time (MTT). CBV reflects the amount of blood present in a given amount of tissue at a given time. It can be estimated from the area under the fitted curve, whereas CBF is computed by the ratio of CBV to MTT. Often in the literature these terms, may be prefixed with the word “relative” (rCBV, rCBF and rMTT), as their absolute quantification is difficult, due to the non-linear relationship between signal change and gadolinium contrast. Thus, it is usually preferable to find a ratio between the ipsilateral and contralateral sides for each hemodynamic parameter [13].

DSCI is associated with certain limitations. First, in tumor cases like high-grade gliomas and meningiomas, where the BBB is disrupted or completely absent, CBV values may be grossly miscalculated. Pre-bolus contrast agent administration has been proposed in order to diminish T1 effects that might result from agent extravasation [40,41]. Secondly, dynamic perfusion MR images are extremely sensitive to structures that induce strong magnetic field inhomogeneities, such as calcium, blood products and areas near brain-bone-air interfaces. One proposed way to reduce these artifacts is to decrease slice section, however this will reduce the signal-to-noise ratio and slice coverage as well [39]. If the tumor cannot be sufficiently covered, the interslice gap can be increased. Nevertheless, this may cause the missing out of small vascular areas, which can however be missed even with thicker slices, due to volume averaging [39]. Lastly, it has to be taken into account that there is a possibility of contrast agent implications. In that case, Arterial Spin Labelling (ASL) has been reported as a promising non-invasive perfusion technique, as it requires no exogenous contrast administration and offers high image quality and quantitative perfusion maps of tissues [42].

## Differential diagnosis of cerebral tumors using DWI, DTI and DSCI

Accurate brain tumor diagnosis plays an essential role in the selection of the optimum treatment strategy, as the nature of the tumor and the grade defines the therapeutic approach. Despite the utilization of advanced MRI techniques, such as DWI/DTI and DSCI, tumor characterization and grading is in some cases a challenging process. The parameters extracted from these techniques provide useful information in a microscopic level however their accurate interpretation is not always straightforward as diffusion and perfusion similarities exist between pathologies, and one should be very careful in correctly combining and evaluating all the available MR data.

### Gliomas

**Figure 1:**
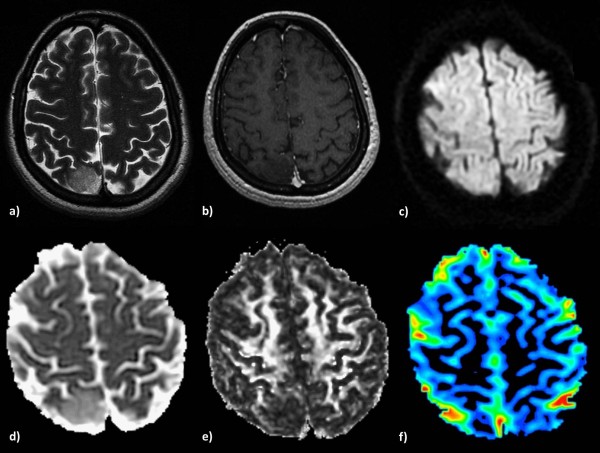
**Low-grade glioma in a 55-year-old woman. a)** High signal intensity on a T2-weighted image, **b)** no contrast enhancement on a 3D-SPGR image and **c)** an isointense signal on a diffusion-weighted image. The lesion shows increased ADC **(d)**, lower FA **(e)** and no significant perfusion (**f)** on the corresponding parametric maps.

**Figure 2:**
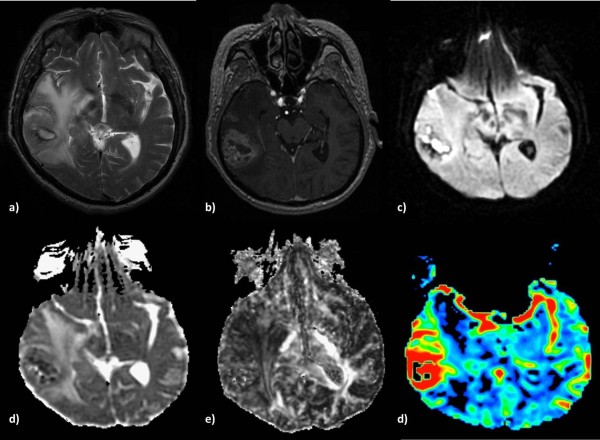
**Anaplastic Astrocytoma in a 71-year-old man. a)** T2-weighted image shows increased signal intensity with peritumoral edema, **b)** heterogeneous contrast enhancement on a post-contrast 3D-SPGR image and **c)** restricted diffusion in the solid portion of the tumor. The lesion is hypointense on the ADC map **(d)**, presents low FA **(e)** and increased perfusion on the rCBV map **(f)**.

**Figure 3:**
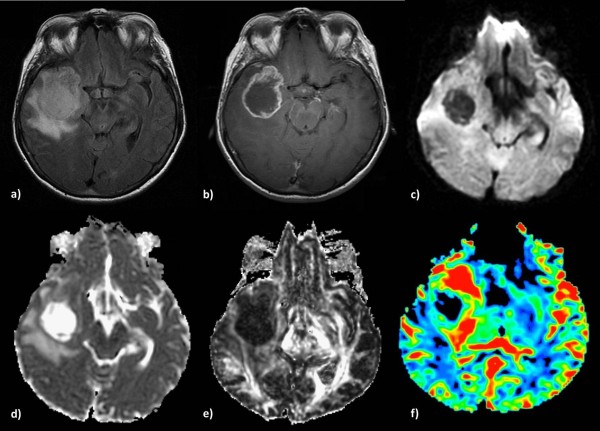
**Glioblastoma multiforme in a 65-year-old woman.** Axial T2-weighted **(a)** and T1-weighted post contrast **(b)** images demonstrate a right temporal lesion with surrounding edema and ring-shaped enhancement. On the DW-image the lesion presents low signal intensity **(c)** resulting in higher intratumoral ADC **(d)**, lower intratumoral FA **(e)**, and high peritumoral rCBV **(f)**, reflecting tumor infiltration in the surrounding parenchyma.

Gliomas represent the most common cerebral neoplasms and the preoperative assessment of their grade is important for therapeutic decision-making. Gliomas arise from supporting glial cells in the brain and the predominant cell type determines the pathological classification. Low-grade gliomas (LGG) consist of Grade I which progress very slowly over time and are usually considered to be benign, and of Grade II that present nuclear atypia, however cellularity and vascularity is low and normal brain is mixed in with the tumor [43]. Depending on their cell origin they may be termed as oligodendroglioma, astrocytoma or mixed type. On conventional MR images LGG present a homogenous structure whereas contrast enhancement and peritumoral edema is usually uncommon (Figure 1) [44]. High-grade gliomas (HGG) consist of Grade III and Grade IV glial tumors. Grade III present mitoses and anaplasia, and their most common subtype is anaplastic astrocytoma (AA) (Figure 2), whereas Grade IV gliomas are characterized by increased cellularity and vascularity with extended necrosis, and are usually termed as glioblastoma multiforme (GBM) (Figure 3). High-grade gliomas present heterogeneous contrast enhancement patterns, necrotic or cystic areas, haemorrhage and infiltrative edema. Nonetheless, the imaging characteristic of these two main glioma categories are not always grade-specific, as in some cases low-grade gliomas may show similar morphological features to high-grade gliomas and the latter may present relatively benign imaging findings [3,45]. Hence, these imaging similarities may potentially lead to inaccurate tumor staging based on conventional MRI alone.

Studies in the literature regarding the contribution of DWI metrics in the differentiation of lower and higher glioma grades have been ambiguous. Due to their cellular structure LGG usually present higher ADC values compared to HGG [44,46,47], however in many cases there is an overlapping between the ADC values of both groups. Zonari et al. reported that even though diffusion was higher in LGG, large variations of ADC values existed between the two groups, thus no significant differences were observed [48]. Similar to Zonari et al. previous studies have concluded that DWI metrics, either from the solid part of the tumor or from the peritumoral edema, are inadequate to provide information about the degree of differentiation of glial tumors [49-51]. In the study of Kono et al. the difference of ADC values between glioblastomas and grade II astrocytomas reached statistical significance, however the authors reported that peritumoral neoplastic cell infiltration cannot be revealed using individual ADC values or even by evaluating ADC maps [52]. In the same study an inverse relationship was observed between diffusion and tumor cellularity, where lower ADC values suggested malignant gliomas, whereas higher ADC values suggested low-grade astrocytomas. Nevertheless, the authors concluded that even though ADC values cannot be reliably used in individual cases to differentiate tumor type, a combination of routine image interpretation and ADC, results in a higher diagnostic value [52]. On the other hand, Fan et al. showed that DWI metrics might be useful in the differentiation of non-enhancing gliomas, as ADC values in anaplastic astrocytomas were significantly lower in the solid portions of the tumors compared to LGG [45]. However, in the same study, no differences were observed for ADC in the peritumoral area of these tumor groups (Table 1).

**Table 1:** Published studies regarding glioma grading and the differentiation of metastatic tumors (MT) from high-grade gliomas (HGG)

	**Authors**	**No. patients**	**Area of measurement**	**Technique**	**Diagnostic outcome**
**Glioma Grading**	Kono et al. [52]	17	Intra/Peritumoral	DWI	Intratumoral ADC higher in LGG than GBM
	Kremer et al. [7]	36	Intratumoral	DSCI	rCBV lower in LGG than HGG
	Beppu et al. [57]	31	Intratumoral	DTI	FA lower in LGG than HGG
	Preul et al. [64]	33	Intratumoral	DSCI	rCBV lower in LGG than HGG
	Law et al. [65]	63	Intratumoral	DSCI	rCBV lower in LGG than GBM
	Inoue et al. [55]	41	Intratumoral	DTI	MD higher in LGG than HGG
					FA lower in LGG than HGG
	Fan et al. [45]	22	Intra/Peritumoral	DWI	Intratumoral ADC higher in LGG than AA
	Hakyemez et al. [101]	33	Intratumoral	DSCI	rCBV lower in LGG than GBM
	Stadlbauer et al. [53]	20	Intratumoral	DTI	FA higher in LGG than HGG
	Zonari et al. [48]	105	Intratumoral	DSCI	rCBV lower in LGG than GBM
	Lee et al. [56]	27	Intratumoral	DTI	MD higher in LGG than HGG
	Di Costanzo et al. [63]	36	Intra/Peritumoral	DSCI	Intra/Peritumoral rCBV lower in LGG than HGG
	Rizzo et al. [49]	35	Intratumoral	DSCI	rCBV lower in LGG than HGG
	Senturk et al. [62]	26	Intratumoral	DSCI	rCBV lower in LGG than HGG
	Chen et al. [59]	31	Intra/Peritumoral	DTI	Peritumoral FA higher in LGG than HGG
	Liu et al. [3]	52	Intratumoral	DTI	FA lower in LGG than HGG
	Svolos et al. [47]	73	Intra/Peritumoral	DWI	Intratumoral ADC higher in LGG than HGG
					Peritumoral ADC lower in LGG than HGG
				DSCI	Intra/Peritumoral rCBV lower in LGG than HGG
**MT vs. HGG**	Chiang et al. [92]	12	Intra/Peritumoral	DSCI	Peritumoral rCBV lower in MT than HGG
	Lu et al. [9]	20	Intra/Peritumoral	DTI	Peritumoral MD higher in MT than GBM
	Server et al. [8]	82	Intra/Peritumoral	DWI	Intratumoral ADC lower in MT than GBM
	Pavlisa et al. [97]	40	Intra/Peritumoral	DWI	Peritumoral ADC higher in MT than GBM
	Wang et al. [98]	63	Intra/Peritumoral	DTI	Intra/Peritumoral FA lower in MT than GBM
	Senturk et al. [62]	18	Intra/Peritumoral	DSCI	Peritumoral rCBV lower in MT than GBM
	Hakyemez et al. [1]	48	Intra/Peritumoral	DSCI	Peritumoral rCBV lower in MT than GBM
	Lee et al. [99]	73	Intra/Peritumoral	DWI	Peritumoral ADC higher in MT than GBM
	Wang et al. [10]	51	Intra/Peritumoral	DTI	Intra/Peritumoral FA lower in MT than GBM
				DSCI	Peritumoral rCBV lower in MT than GBM
	Server et al. [94]	61	Intra/Peritumoral	DSCI	Peritumoral rCBV lower in MT than GBM
	Tsougos et al. [11]	49	Intra/Peritumoral	DSCI	Peritumoral rCBV lower in MT than GBM
	Lehmann et al. [93]	24	Peritumoral	DSCI	rCBV lower in MT than GBM.
	Svolos et al. [47]	71	Intra/Peritumoral	DTI	Peritumoral FA lower in MT than HGG
				DSCI	Peritumoral rCBV lower in MT than HGG

The number of patients, the investigated tumor areas, the most useful imaging techniques and the diagnostic outcome for each case are summarized in the Table.

Conflicting results are also reported in the literature regarding the ability of DTI parameters to discriminate between LGG and HGG. Studies have shown that MD measured in the intratumoral area [9,53,54] or in the peritumoral area [9,54] cannot be used as a predictor of lower and higher glioma grades. Contrary to these findings, Inoue et al. have reported that MD within grade I gliomas was significantly higher compared to grade III and grade IV gliomas respectively, but no differences were observed in MD values between grade II and grade III gliomas [55]. However, regarding HGG that may present relatively benign imaging findings, such as absence of contrast enhancement, Lee et al. showed that MD is significantly lower in the non-enhancing regions of HGG compared to LGG [56]. Similar tendencies were observed in the study of Liu et al. although these differences did not reach statistical significance [3].

Glioma grading and tumor infiltration have been also investigated in terms of FA measurements. Studies of the relationship between DTI and histological malignancy of gliomas showed that FA can distinguish HGG from LGG, thus be useful in deciding the surgical strategy or the selected site of stereotactic biopsy [53,55,57]. Inoue et al. reported that FA is significantly higher in HGG than LGG and a cut-off value of 0.188 was proposed between the two groups [55]. In the same study, a positive correlation of FA with cell density of gliomas was observed in agreement with the results from Beppu et al. [57]. On the contrary, Stadlbauer et al. reported a negative correlation between FA and glioma cellularity, however the authors concluded that FA is a better indicator than MD for the assessment and delineation of different degrees of pathologic changes in gliomas [53]. Additionally, in agreement with their non-infiltrating nature, higher FA values have been observed in the periphery of LGG indicating the presence of well-preserved fibers in the area, in contrast to HGG where peritumoral tracts are disarranged or disrupted [58,59]. Even in the case of non-enhancing HGG, FA has been reported to provide useful information regarding differentiation from LGG [3]. Liu et al. observed that the mean and maximal FA values were significantly lower in LGG, and proposed a cut-off value of 0.129 between the two groups. Moreover, the authors showed that diagnostic accuracy improves if these two parameters are combined rather than evaluated separately, concluding that this may be useful in the preoperative grading of supratentorial non-enhancing gliomas [3]. Similar to Liu et al., Ferda et al. reported that the co-evaluation of FA maps and contrast enhancement patterns may improve the possibility of distinguishing among lower and higher gliomas grades [60]. Nonetheless, controversies regarding the contribution of FA still exist in the literature, as a number of studies conclude that the utility of DTI metrics in the preoperative grading of enhancing and non-enhancing gliomas, regardless of the area of measurement, is still limited [9,54,56,58,59] (Table 1).

Contrary to DWI and DTI, DSCI has been shown to provide a robust differentiation between LGG and HGG. The difference in vascular morphology and degree of angiogenesis, which are important histopathological factors determining the malignancy and grade of glial tumors, can be reflected in the rCBV values measured in these tumors [39,61]. Due to their low vascularity, LGG present no or minimally increased rCBV in the intratumoral area compared to the contralateral normal side, and significantly lower mean rCBV when compared to higher glioma grades. Anaplastic astrocytomas have higher rCBV values than LGGs but lower than GBMs, which are the most hypervascular among all gliomas. The characteristic difference in the underlying vascularity between low and high glioma grades has been stressed by a number of previous studies [7,47-49,61-67]. Although, different mean rCBV values have been proposed for each glioma group, all of the studies agree that there is a progressive increase in rCBV from lower to higher glioma grades, which is highly correlated to the microvascular density of each grade. However, it should be noted that gliomas comprise a relative heterogeneous group of tumors, thus overlapping in rCBV values among different grades might be observed. Low-grade oligodendrogliomas or even pilocytic astrocytomas, which are considered benign, have been histologically verified to exhibit increased angiogenesis and elevated rCBV values, comparable to those of malignant gliomas [66,68-71]. This aspect (histological type) should be taken into account when glioma-grade comparisons are conducted (Table 1).

The comparison between non-enhancing HGG and LGG in terms of perfusion measurements has yielded conflicting results, as Liu et al. observed similar rCBV ratios between the two glioma groups [3], whereas Fan et al. and Maia et al. reported that rCBV values from the non-enhancing regions of HGG were significantly higher than LGG but no threshold was proposed between the groups [45,72]. Fan et al. observed significant differences in peritumoral rCBV values as well between low- and high- grade non-enhancing gliomas [45]. Regardless of these variations the key point is that rCBV presents in general a strong and positive correlation to glioma grade, thus rCBV may constitute an important marker of tumor angiogenesis and malignancy. Nevertheless, despite the diagnostic usefulness of DSCI alone, it should be stressed that in all cases, perfusion measurements and rCBV maps must be always co-evaluated with conventional imaging and clinical findings for accurate glioma characterization and grading [39,67].

### Meningiomas

**Figure 4:**
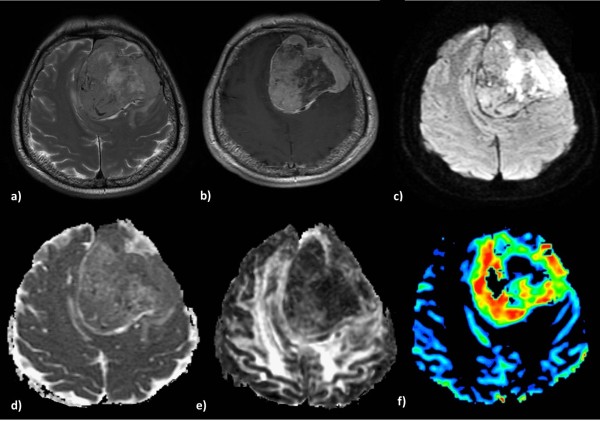
**A typical Meningioma in a 60-year-old man.** Axial T2-weighted **(a)** and postcontrast T1-weighted **(b)** images demonstrate a large heterogeneous enhanced left frontal mass with an intense mass effect. The lesion presents areas of restricted diffusion **(c)**, isointensity on the ADC map **(d)**, hypointensity on the FA map **(e)** and elevated blood volume **(f)**.

Meningiomas are the most common extra-axial cerebral tumors, originate from the dura matter and their characteristic location enables their relatively straightforward diagnosis. Grade I meningiomas are benign and usually full recovery is achieved with surgical resection. Grade II (atypical) and Grade III (malignant) meningiomas are less common but more aggressive than Grade I, thus they are more likely to recur even after complete resection [66]. According to WHO classification the differences between benign and atypical/malignant meningiomas relate to the number of mitoses, cellularity, and nucleus-to-cytoplasm ratio as well as their histologic patterns [73]. Regardless of the grade, meningiomas are highly vascular lesions that derive blood mostly from meningeal arteries. Their tumor capillaries present complete lack of the BBB, thus increased contrast leakage and permeability is observed on perfusion images. Conventional MR imaging provides useful information regarding their localization and morphology, however in cases which meningiomas present atypical imaging findings mimicking high-grade tumors, their histologic grading is of significant importance for beneficial treatment planning (Figure 4).

The usefulness of diffusion and perfusion techniques either in meningioma grading or in the differentiation of benign and atypical/malignant subtypes has been previously systematically investigated. Previous studies in the literature have shown that DWI and DTI metrics from the intratumoral region are useful in meningioma grading [5,74-76] (Table 2). Lower ADC and higher FA values have been reported for atypical/malignant meningiomas compared to benign, indicating an inverse relationship between water diffusion and malignancy. The increased mitotic activity, necrosis, the high nucleus-to-cytoplasm ratio as well as the uninterrupted patternless cell growth present in high-grade meningiomas [77], leads to restricted water diffusion, which is reflected in the related diffusion parameters. On the contrary, benign meningiomas show histologically lack of coherent organization, as they consist of oval or spindle-shaped neoplastic cells that form whorls, fascicles, cords, or nodules, forcing water molecules to move in a relatively isotropic way [78]. DSCI metrics have been also reported useful in meningioma grading, as in the study of Zhang et al., malignant meningiomas had higher rCBV ratios in their periphery compared to benign. However, no differences were observed in intratumoral rCBV measurements most probably due to the characteristic inherent hypervascularity of all grades [79]. Regarding the differentiation between subtypes of the same grade, FA was significantly different between subtypes of benign meningiomas [80], whereas ADC and rCBV did not contribute either in benign or malignant subtype discrimination [80,81]. Nevertheless, controversies still exist in the literature, as a number of previous studies conclude that diffusion and perfusion quantification, derived either from the tumor [46,79,82-84], or from the peritumoral edema [5,75], cannot provide significant information for meningioma grading (Table 2).

**Table 2:** Published studies regarding different tumor comparisons

**MNG vs. HGG**	**Authors**	**No. patients**	**Area of measurement**	**Technique**	**Diagnostic outcome**
	Kremer et al. [7]	37	Intratumoral	DSCI	rCBV higher in MNG than HGG
	Tropine et al. [54]	22	Intra/Peritumoral	DTI	Intratumoral MD lower in MNG than HGG
					Intratumoral FA higher in MNG than HGG
	Hakyemez et al. [5]	49	Intratumoral	DSCI	rCBV higher in MNG than HGG
	De Belder et al. [4]	35	Intra/Peritumoral	DTI	Intratumoral ADC lower in MNG than HGG
					Intratumoral FA higher in MNG than HGG
					Peritumoral FA higher in MNG than HGG
	Svolos et al. [47]	77	Intra/Peritumoral	DWI	Intra/Peritumoral ADC lower in MNG than HGG
				DTI	Intra/Peritumoral FA higher in MNG than HGG
				DSCI	Peritumoral rCBV lower in MNG than HGG
**MNG vs. MT**	Kremer et al. [7]	21	Intratumoral	DSCI	rCBV higher in MNG than MT
	Hakyemez et al. [5]	48	Intratumoral	DSCI	rCBV higher in MNG than MT
	Toh et al. [88]	26	Peritumoral	DTI	MD lower in MNG than MT
					FA higher in MNG than MT
	Svolos et al. [47]	42	Intra/Peritumoral	DWI	Intra/Peritumoral ADC lower in MNG than MT
				DTI	Intra/Peritumoral FA higher in MNG than MT
**PCL vs. HGG**	Guo et al. [105]	28	Intratumoral	DWI	ADC lower in PCL than HGG
	Cho et al.[111]	29	Intratumoral	DSCI	rCBV lower in PCL than HGG
	Kremer et al. [7]	32	Intratumoral	DSCI	rCBV lower in PCL than GBM
	Hartmann et al. [112]	24	Intratumoral	DSCI	rCBV lower in PCL than GBM
	Yamasaki et al. [46]	44	Intratumoral	DWI	ADC lower in PCL than GBM
	Calli et al. [6]	25	Intratumoral	DWI	ADC lower in PCL than GBM
				DSCI	rCBV lower in PCL than GBM
	Hakyemez et al. [101]	31	Intratumoral	DSCI	rCBV lower in PCL than HGG
	Rollin et al. [109]	10	Intra/Peritumoral	DSCI	Intra/Peritumoral rCBV lower in PCL than HGG
	Toh et al. [106]	20	Intratumoral	DTI	MD and FA lower in PCL than GBM
	Kinoshita et al. [107]	14	Intratumoral	DTI	MD lower in PCL than HGG
	Server et al. [8]	64	Intra/Peritumoral	DWI	Intratumoral ADC lower in PCL than HGG
	Liao et al. [38]	28	Intratumoral	DSCI	rCBV lower in PCL than HGG
	Bendini et al. [110]	23	Intratumoral	DSCI	rCBV lower in PCL than HGG
	Wang et al. [10]	42	Intra/Peritumoral	DTI	Intratumoral MD and FA lower in PCL than GBM
					Peritumoral FA lower in PCL than GBM
				DSCI	Intra/Peritumoral rCBV lower in PCL than GBM
**PCL vs. MT**	Kremer et al. [7]	16	Intratumoral	DSCI	rCBV lower in PCL than MT.
	Cho et al. [111]	15	Intratumoral	DSCI	rCBV lower in PCL than MT.
	Yamasaki et al. [46]	37	Intratumoral	DWI	ADC lower in PCL than MT
	Hakyemez et al. [101]	30	Intratumoral	DSCI	rCBV lower in PCL than MT
	Server et al. [8]	28	Intra/Peritumoral	DWI	Intratumoral ADC lower in PCL than MT
	Wang et al. [10]	41	Intra/Peritumoral	DTI	Intratumoral MD lower in PCL than MT
**Abscess vs. Cystic/Necrotic Tumor**	Hartmann et al. [117]	17	Intratumoral	DWI	ADC lower in abscess than other tymors.
	Chan et al. [115]	12	Intra/Peritumoral	DWI	Intratumoral ADC lower in abscess than other tumors
					Peritumoral ADC higher in abscess than other tumors
				DSCI	Intra/Peritumoral rCBV lower in abscess than other tumors
	Chang et al. [2]	26	Intratumoral	DWI	ADC lower in abscess than other tumors
	Lai et al. [118]	14	Intratumoral	DWI	ADC lower in abscess than other tumors
	Nadal-Desbarats et al. [119]	26	Intratumoral	DWI	ADC lower in abscess than other tumors
	Holmes et al. [123]	8	Intratumoral	DSCI	rCBV lower in abscess than other tumors
	Hakyemez et al. [101]	55	Intratumoral	DSCI	rCBV lower in abscess than other tumors
	Nath et al. [120]	53	Intratumoral	DTI	MD lower and FA higher in abscess than other tumors
	Reiche et al. [121]	17	Intratumoral	DWI	ADC lower in abscess than other tumors
				DTI	MD lower and FA higher in abscess than other tumors

The number of patients, investigated tumor areas, most useful imaging techniques and the diagnostic outcome for each case are summarized in the Table.

Note- MNG = Meningioma, HGG = High-grade glioma, MT = Metastatic Tumor, PCL = Primary Cerebral Lymphoma.

Differences between meningiomas and gliomas have been also investigated in terms DWI/DTI and DSCI metrics. Similarities in diffusion properties have been reported between meningiomas and low-grade gliomas [9,52] however in the study of Tropine et al. significant differences in MD and FA were observed between the two groups [54]. Low-grade gliomas had higher MD and lower FA values in the intratumoral region compared to meningiomas, which might be attributed to their lower tumor cellularity. No differences were observed in the related parameters of the peritumoral edema, most probably due to the non-infiltrating nature of both tumor types [54].

Regarding meningiomas that may present atypical imaging findings and might be misdiagnosed as high-grade gliomas the results in the literature are mixed. A number of studies suggest that diffusion and perfusion quantification can be helpful in correctly characterizing these lesions and thus aid treatment planning (Table 2). Lower ADC and higher FA values as well as elevated rCBV ratios have been observed in the solid portion of meningiomas compared to HGG [4,5,7,54]. The differences in diffusion properties between these two tumor types indicate a higher level of fibrous organization in meningiomas compared to HGG, which present a more incoherent cellular structure [4]. Contrary to these observations the contribution of DWI/DTI metrics in the preoperative differentiation of these two groups has been reported insignificant [8,9]. Regarding perfusion, meningiomas have been characterized as particularly hypervascular lesions hence increased rCBV values are expected. However, despite their higher rCBV values significant overlapping has been observed between meningiomas and high-grade glial tumors not allowing their distinct differentiation [62]. This overlapping may be due to the highly leaky and permeable capillaries of meningiomas leading to grossly over-or-underestimated rCBV measurements, and should be taken into consideration when meningiomas are compared to other tumor types [39]. Furthermore, research has been also conducted to identify differences related to the characteristic nature of these lesions, which is infiltration vs. non-infiltration, however the results obtained were insignificant [4,8,9,52,54,85,86].

Apart from high-grade gliomas, meningiomas that present atypical imaging findings may resemble solitary metastatic tumors as well. Previous studies have shown that intratumoral rCBV measurements may provide significant differentiation among the two tumor groups even though metastases present increased vasculature on perfusion images similar to meningiomas [5,7] (Table 2). In the study of Hakyemez et al. metastatic tumors had elevated rCBV values, but these values were significantly lower compared to meningiomas [5]. These results are in disagreement with the study of Lui et al., in which the authors conclude that the differentiation of the two tumor groups through perfusion measurements is not feasible [87]. Similarities of the diffusion profiles in meningiomas and metastases have been also reported [8,9,52,86]. Based on these studies, diffusion and anisotropy changes, either from the solid region or the periphery of the tumor, are inadequate to distinguish meningiomas from metastases. These findings may be explained by the fact that atypical/malignant meningiomas often present a heterogeneous cellular structure, with necrotic and cystic portions, thus inducing unhindered water diffusion comparable to that of metastatic tumors. Furthermore, as non-infiltrating lesions, their surrounding edema is purely vasogenic, and cannot provide distinct information in terms of DWI/DTI measurements. Nevertheless, regarding the nature of their surrounding edema, a previous study demonstrated that ADC and FA values are significantly different between meningiomas and metastases [88]. Based on this study, as the mechanisms of edema formation in metastatic brain tumors and meningiomas may derive from different factors, the classification of peritumoral edema in purely vasogenic and infiltrating might not be sufficient. Therefore the authors suggest that DTI could potentially identify subtle differences in the ‘purely vasogenic’ edema associated with different tumor groups [88].

### Cerebral metastases

**Figure 5:**
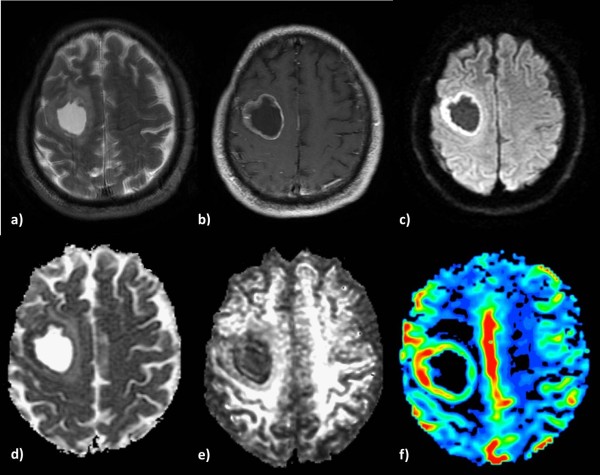
**Intracranial lung metastasis in a 68 year-old-man. a)** T2-weighted image, **b)** ring-shaped enhancement on a T1-weighted post contrast edema, **c)** restricted diffusion in the periphery of the tumor. Increased intratumoral ADC **(d)**, decreased FA **(e)** and elevated perfusion in the peripheral solid part of the lesion **(f)**.

The incidence of cerebral metastases is rapidly increasing and approaches almost 50% of all brain tumors in adults [89]. The most common primary cancers that metastasize to the brain are lung, breast, colon, malignant melanoma and gastro-intestinal cancers [90]. Metastatic tumors spread into the central nervous system via hematogenous routes and induce neovascularization as they grow and expand, however their capillaries are leaky due to the lack of a well-developed BBB with tight junctions [67]. The differentiation of metastases from other malignant tumors on conventional MRI is usually straightforward due to the clinical history of the patient or the existence of multiple well-circumscribed lesions. However, the occurrence of a solitary enhancing lesion without the knowledge of a primary tumor complicates differential diagnosis, because it may present similar imaging characteristics and contrast enhancement patterns like those of high-grade gliomas (Figure 5). Hence, the accurate characterization of these lesions is clinically important as medical staging, surgical planning and therapeutic approach differ significantly between these tumor entities [90,91].

The differentiation of metastases from primary high-grade gliomas has been extensively investigated in the literature as they represent a common differential diagnostic problem. DSCI has been suggested as a useful technique in discriminating the two tumor groups, based on differences in the underlying pathophysiology of their peritumoral area [1,10,11,62,92-94] (Table 1). High-grade gliomas are characterized by the ability to recruit and synthesize vascular networks for further growth and proliferation. Hence, tumor cells are expected to be present in their periphery along with increased edema concentration (infiltrating edema). On the other hand, metastatic brain tumors arise within the brain parenchyma and usually grow by expansion, displacing the surrounding brain tissue, and with no histologic evidence of tumor cellularity outside the contrast-enhanced margin of the tumor (pure vasogenic edema) [66,95]. Therefore, because of this difference increased rCBV ratios have been measured in the surrounding edema of high-grade gliomas compared to solitary metastases, enabling the robust differentiation of these two tumor groups (Table 1). Regarding their intratumoral region, no differences have been observed in the measured rCBV ratios, most probably due to high vascularity and abnormal capillary permeability of both tumor types [1,5,6,10,11,62,92]. Apart from rCBV measurements, two additional hemodynamic variables have been also evaluated regarding the differentiation of high-grade gliomas and secondary brain tumors; the peak height of maximal signal intensity drop and the percentage of signal intensity recovery after the end of first pass of gadolinium bolus. The peak height has been shown to correlate with rCBV and thus reflect total capillary volume, whereas the percentage of signal intensity recovery reflects the alteration in capillary permeability [96]. In the study of Cha et al., the authors showed that the average peak height was increased in the peritumoral edema of GBMs compared to metastatic tumors [96]. Furthermore, metastatic tumors presented significant reduction in the average percentage of signal intensity, in both regions of interest, compared to glioblastomas.

Increased ADC and lower FA have been reported both in metastases and high-grade gliomas, which might be attributed to their heterogeneous cellular structure. Furthermore, the presence of increased edema in their periphery, even though of different nature (vasogenic versus infiltrating), does not provide any significant information that may allow a distinct differentiation between the two tumor groups. Hence, a large number of studies in the literature have concluded that the contribution of DWI and DTI metrics, either in the tumor or the peritumoral area, is still limited [6,10,11,46,91,97,98]. Contrary to these observations there have been studies showing that the diffusion profiles of high-grade gliomas and metastases, either within or around the tumors, differ and that diffusion measurements may be indicative for tumor discrimination [8,9,92,97-99] (Table 1). Based on these studies, high-grade gliomas present elevated FA in the intratumoral and peritumoral region compared to metastases, whereas the latter present increased ADC values in their periphery. Restricted diffusion in high-grade gliomas might be explained by higher cellularity in their solid part compared to metastases, as well as by the presence of tumor cells in their periphery due to their infiltrating nature [11,100]. On the other hand, metastases have been associated with increased edema concentration, as a result of their leaky tumor capillaries, leading to higher ADC values in the peritumoral parenchyma [9,92]. Nevertheless, it is evident that the utility of DWI and DTI in preoperative differentiation of solitary metastatic tumors from high-grade gliomas remains controversial and further studies are required for accurate tumor assessment.

Differentiation of secondary tumors from low-grade gliomas is usually straightforward, due to absence of contrast-enhancement in low-grade gliomas and the presence of minimal or no peritumoral edema around them. However, if low-grade glial tumors do not present typical imaging and contrast-enhancement findings, ADC in their intratumoral region has been reported significantly higher than in metastases, allowing their distinct differentiation [52]. In the same study no differences were observed for peritumoral ADC values.

Discrimination between different types of metastases in terms of perfusion and diffusion measurements has also been investigated [49,101]. Hakyemez et al. searched for potential perfusion changes in lung and breast metastases however the authors concluded that the difference in rCBV values showed no statistical significance [101]. Similar results were observed in the study of Rizzo et al., even though multiple types of metastases were examined such as from primary melanoma, lymphoma, breast, lung and gastrointestinal cancer. ADC values and rCBV ratios between all types showed wide variability with considerable overlapping and no statistical differences, with the lowest rCBV ratio seen in metastases from primary lymphoma [49]. Nevertheless, as DSCI imaging is sensitive in detecting abnormal perfusion changes in tissues, it would be of great interest to examine a larger number of metastases presenting hypo-or hypervascularity in order to correctly classify them.

### Primary cerebral lymphoma

**Figure 6:**
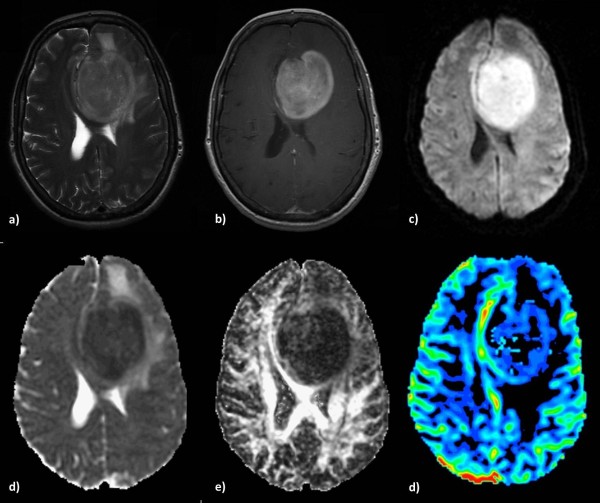
**Primary Cerebral Lymphoma in a 59-year-old-woman. a)** High signal intensity with peritumoral edema on a T2-weighted image, **b)** intense contrast-enhancement on a T1-weighted post contrast image and **c)** hyperintensity on a DW image**.** Decreased intratumoral ADC **(d)** and FA **(e).** The rCBV map shows moderate perfusion within the lesion **(f).**

The incidence of Primary Cerebral Lymphomas (PCL) has been substantially increased over the last three decades and currently accounts for about 6% of all cerebral tumors. PCLs are aggressive neoplasms with increased incidence in immunocompetent as well as immunocompromised patients [102]. Lymphomas tend to be round or oval lesions in appearance and peritumoral edema is typically identified around them. Because these tumors are usually infiltrative in nature and not encapsulated, the borders of the MR signal change may not necessarily reflect the true tumor margin [103]. One of the most significant histopathologic characteristics of a PCL is the angiocentric growth with neoplastic cells forming multiple, thick layers around blood vessels. Tumor invasion of endothelial cells in the perivascular spaces and within the vessel walls can be often observed, however neoangiogenesis is not a prominent feature [10,39,104]. Furthermore, PCLs present a remarkable contrast enhancement on conventional MR images due to the complete absence of BBB (Figure 6) [67]. However, because of their diffuse infiltrative growth in some cases it is difficult to distinguish them from high-grade gliomas and solitary metastases based on conventional MRI findings alone [101]. The preoperative differentiation of PCLs from other high-grade malignancies is important as pre-surgical staging, intraoperative management, and postoperative treatment differ significantly between these tumors [38].

Under this perspective, many studies have been performed using advanced MRI techniques in order to differentiate PCLs from glioblastomas and metastases. DWI and DTI have been helpful in distinguishing these tumor groups [6,10,46,105-108] (Table 2). As highly cellular tumors, PCLs have a relatively decreased amount of extracellular space, causing a restriction to free water diffusibility. Hence, PCLs have been reported with significantly lower ADC values than HGGs and metastases. Similar to ADC, lower FA values have been also observed in PCLs compared to high-grade malignancies [10,106]. This is a conflicting finding regarding the high cellularity of PCLs, however the relationship of FA and tumor cellularity still remains controversial, as both positive and negative correlations have been reported [53,55,57]. Despite the encouraging results regarding diffusion quantification in the discrimination of PCLs from both metastatic tumors and high-grade gliomas, there have been studies in the literature concluding that the contribution of DWI and DTI is insignificant in both the intratumoral [6,8,49,107,109] and peritumoral area [8,10] of these lesions.

On the other hand DSCI metrics have showed a substantial ability to differentiate between these tumor groups (Table 2). Densely contrast-enhanced PCLs present lower rCBV values compared to HGG and metastases [6,7,10,38,49,101,109-113]. Strong enhancement without CBV increment in lymphomas is attributed to the BBB destruction without neovascularization, contrary to the marked contrast enhancement with increased vascularity in HGG and metastatic tumors [67]. Furthermore, a higher signal drop in the intensity-time curve has been observed for PCLs compared to high-grade malignancies, followed by a significant increase in signal intensity above the baseline [38,111]. This finding is considered to be the result of the massive leakage of contrast agent into the interstitial space of PCLs. The rCBV ratio has been also reported significantly lower in their periphery compared to glioblastomas, most probably due to higher infiltrations of the latter however no differences were observed in peritumoral perfusion changes between PCLs and metastases [10].

The differentiation of PCLs from low-grade gliomas does not comprise a diagnostic dilemma and is usually direct, because of the intense contrast-enhancement and peritumoral edema of PCLs compared to LGG. However, if LGG lack their conventional imaging findings, ADC in their intratumoral region has been reported higher than PCLs whereas FA values are lower respectively [107]. Bendini et al. observed significant differences in perfusion properties as well between the two groups however these findings are in disagreement with the ones reported by Kremer et al. [7,110].

Overall, the higher cellularity, the absence of neoangiogenesis and the different patterns of contrast leakage lend PCLs with characteristic diffusion and perfusion features, which enable their differentiation from glial tumors and solitary metastases.

### Intracranial abscesses

Brain abscesses are focal lesions caused by an infectious process of micro-organism or pathogens which produce an area of focal cerebritis leading to accumulation of purulent exudates in the brain tissue. A capsule of collagenous substance begins to grow and encapsulate the purulent focus [114,115]. Pus is a highly viscous, thick, mucoid fluid consisting of inflammatory cells, bacteria, proteoneous exudate and fibrinogen. In some cases (especially in the capsule stage), radiologic diagnosis of cerebral abscesses may be challenging due to the variable appearance of these lesions secondary to different offending microbes and different stages of manifestation [67]. On conventional MRI, abscesses present increased signal intensity on T2-weighted images with associated peritumoral edema, increased signal intensity on DW images and ring-shaped contrast enhancement. These features are non-specific, and cystic or necrotic tumors (glioblastomas and solitary metastases) may contain pus, thus complicating their direct differentiation from cerebral abscesses [67,101,115].

DWI and DTI metrics have been proven beneficial in differentiating between abscesses and other cystic lesions [2,115-121]. Based on these studies, lower ADC values are observed in the central cavity of abscesses compared to glioblastomas and metastases (Table 2). This is attributed to the high viscosity and cellularity of pus, which results in substantially restricted diffusion, contrary to the low viscosity in the cystic or necrotic areas of tumors, that facilitates free diffusion and results in higher ADC values [117,118,121]. Significant differences in ADC values have been also observed between the capsular wall of abscesses and the peripheral tumor wall. Chan et al. reported that the capsular wall was hypointense on DW images and higher ADC values were measured in the area, compared to the hyperintese tumor wall, associated with lower ADC values [115]. The authors suggested that inflammation induced increased extracellular fluid accumulation in the abscess wall, thus water diffusion was unhindered. On the contrary, the higher cellularity in the peripheral wall of the tumors, due to closely packed malignant cells, resulted in restricted diffusion [115]. Furthermore, these intra-cavity histological differences are reported to be responsible for the higher FA values measured in cerebral abscesses compared to cystic or necrotic tumors. This may be attributed to the more organized structure of inflammatory cells, owing to cell adhesion secondary to expression of various cell adhesion molecules on the surface of inflammatory cells [120,122].

The role of DSCI metrics in the differentiation of infectious abscesses from other tumor types has been proven significant as well. Glioblastomas and metastases demonstrated higher rCBV ratios than abscesses, either in the intratumoral [101,115,123] or the peritumoral area [115] (Table 2). Low vascularity and decreased neoangiogenesis characterizes brain abscesses compared to hypervascular high-grade tumors. Therefore, the difference in capillary density between these tumors enables their distinct discrimination. Furthermore, perfusion measurements may be also helpful in the differentiation of various central nervous infections. Cha et al. reported significantly lower rCBV values for herpes and toxoplasmosis encephalitis compared to bacterial abscesses, which demonstrated areas of increased perfusion [67].

Overall, the high viscosity and cellularity of intracranial abscesses are the main biological factors that define the diffusion and perfusion characteristics of these lesions. DWI, DTI and DSCI metrics can identify these distinct characteristics and provide a direct differentiation from cystic and necrotic tumors.

## Parametric combination - future perspectives

The role of MRI in the detection of cerebral tumors has been well established. The excellent soft tissue visualization and the great variety of imaging sequences, which are the main advantages of conventional MRI, are in many cases non-specific for the assessment of tumor grading. Hence, advanced techniques, like DWI, DTI and DSCI, which are based on different contrast principles, have been used in the clinical routine to improve diagnostic accuracy. Furthermore, over the years MR systems have evolved from imaging modalities to advanced systems that produce a variety of numeric parameters. This variety of quantitative information derived from these techniques provides significant structural and functional information in a cellular level, highlighting aspects of the underlying brain patho-physiology. Exploiting these advanced technological and imaging capabilities of MR systems is of great importance to optimize tumor diagnosis and treatment.

Hence, the contribution of advanced techniques to the preoperative assessment of tumor grade and infiltration has been proven useful in many cases [2-6,124], however despite the variety of the available MR data no single technique can provide a robust tumor characterization. Additionally, the reported results in the literature are conflicting and complicate even further clinical decision-making [7-11]. These controversies reflect the complex underlying pathophysiologic mechanisms, which are present in cerebral lesions and prevent in some cases the clear discrimination between tumors.

Tumor cellularity and vascularity are usually the most critical elements in the determination of tumor grade and prognosis. These two factors can be quantified through diffusion and perfusion metrics, however as they are closely correlated, their evaluation and interpretation is difficult on the basis of individual numeric parameters. Conventional methods of data analysis, such as searching for statistical significances of the related parameters between different tumor groups may be efficient in some cases. However, in more demanding diagnostic problems like tumors that have similar patho-physiological profiles, their efficiency might be limited. The last few years, diagnostic interest has been focused on the combination of different parameters provided by advanced MRI techniques, and the incremental diagnostic and predictive value that multi-parametric analysis may yield. Different methods of data-analysis have been evaluated, such as Logistic Regression (LR) and Receiver Operating Characteristic (ROC) analysis [10,48,94,125] as well as more sophisticated techniques like machine learning algorithms [126-128], using various parametric combinations.

Server et al. reported that the combination of DWI and Magnetic Resonance Spectroscopic Imaging (MRSI) increased the accuracy of preoperative differentiation of LGG vs. HGG, compared to DWI or MRSI alone [94]. In this study the four factor model, consisting of intratumoral mean ADC and maximum ADC and peritumoral Cho/Cr and Cho/NAA ratios, resulted in 92.5% accuracy, 91.5% sensitivity, 100% specificity, 100% Positive Predictive Value (PPV) and 60% Negative Predictive Value (NPV). Wang et al. investigated if the combination of DTI and DSCI can assist in better differentiation of glioblastomas, solitary brain metastases, and PCLs [10]. The authors showed that the best model to discriminate glioblastomas from non-glioblastomas consisted of ADC and FA from the enhancing region of the tumors and rCBV from the immediate peritumoral region. The accuracy, sensitivity and specificity scored were 93.8%, 89%, and 93% respectively. Additionally, the best model to differentiate PCLs from metastases consisted of ADC from the enhancing regions and the planar anisotropy coefficient (CP) from the immediate peritumoral area. The accuracy, sensitivity and specificity were 90.9%, 77% and 94% respectively [10]. Law et al. compared the diagnostic value of perfusion and metabolic data over conventional MRI alone in glioma grading [124]. The performance of intratumoral rCBV, Cho/Cr and Cho/NAA yielded the highest performance, scoring 93.3% sensitivity, 60% specificity, 87.5% PPV and 75% NPV, whereas the corresponding values for conventional MRI were 72.5%, 65%, 86.1% and 44.1% respectively [124]. Similarly Zonari et al. showed that the differentiation of LGG and HGG is more efficient if DWI, DSCI and MRSI data are combined than evaluated independently [48].

Hence, it seems that multi-parametric analysis may substantially improve diagnostic accuracies over conventional MRI alone, and highlight the underlying pathophysiology. However, it is a quite demanding and time-consuming process, due to the numeric nature of the acquired MR data. Recent studies have reported that machine learning techniques may be used as an automated computer analysis tool, in order to aid tumor diagnosis [126,129-131]. The use of such techniques, allows the manipulation and evaluation of a large amount of quantitative data during clinical practice. A variety of features, such as morphological (e.g. tumor shape and texture) and conventional (e.g. signal intensity) extracted from different MR sequences, have been evaluated with very interesting results [132,133]. Nonetheless, the combination of conventional features with ones extracted from diffusion, perfusion and spectroscopic sequences, or the combination of DWI/DTI, DSCI and MRSI data, has demonstrated increased discrimination accuracies for binary and multiclass classification problems [126,128-131,134]. However, the most important aspect of machine learning techniques is their additional ability to provide predictive outcomes, in contrast to conventional statistical methods, which are limited to producing diagnostic results retrospectively.

Georgiadis et al. investigated the efficiency of combined textural MRI features and MRSI metabolite ratios employing the Support Vector Machine (SVM) algorithm for the discrimination of metastatic tumors from meningiomas [126]. This combination resulted in 92.15% overall accuracy between the two groups and 100% correctly classified meningiomas and metastases cases derived from an independent test set. Hu et al. used one class SVM in order to differentiate radiation necrosis from tumor recurrence in patients with resected GBMs using DWI, DSCI and conventional imaging data [130]. The authors concluded that perfusion and diffusion parameters made a much greater contribution to the discrimination than conventional MRI. Accuracy, sensitivity and specificity were 94.4%, 88.9% and 93.7% respectively. Tsolaki et al. evaluated the diagnostic contribution of SVM, Naïve-Bayes and k-Nearest Neighbor (k-NN) algorithms in the differentiation of glioblastomas and solitary metastases using different combinations of metabolic and perfusion parameters [129]. SVM reached the highest performance in the intratumoral area for the combination of rCBV and NAA/Cr, Cho/Cr and (Lip + Lac)/Cr ratios, whereas in the peritumoral area both SVM and Naïve-Bayes showed high classification performance using NAA/Cr and rCBV as features. Furthermore, the evaluation of an independent test set consisting of glioblastomas and metastases, resulted in one misclassified metastasis case in the intratumoral area by SVM, whereas the same algorithm classified correctly all clinical cases in the peritumoral region [129].

## Conclusion

In conclusion, the characterization of tumoral and peritumoral tissue microstructure, based on water diffusion and perfusion findings, results in increased diagnostic value. Hence, it is evident that the combination of diffusion, perfusion and spectroscopic parameters, either within a statistical model or a classification scheme, should further improve the diagnostic outcome. Logistic regression and ROC analysis may be useful in the characterization and grading of brain tumors using parametric combinations, however the discrimination accuracy and specificity may be further improved, especially for tumors that present similar histo-pathological profiles, if sophisticated machine learning algorithms are used. The additional ability of these algorithms to provide predictive outcomes enables their integration in clinical decision support systems to optimize differential diagnosis. Nevertheless, it should be stressed that regardless of the predicted outcome, one should always take into consideration that final decision-making is not a one-step procedure. The wide range of metabolic, functional and structural data derived from advanced MRI techniques if thoroughly evaluated and combined with other clinical and imaging findings might be the key to optimize diagnosis and treatment.

## Competing interests

The authors declare that they have no competing interests.

## Authors’ contributions

PS conducted a thorough literature research, evaluated and selected the studies that present critical information and drafted the manuscript. EK contributed in the structuring and editing of the manuscript. EK, KT, IF, CK revised the manuscript critically for important intellectual content and contributed in the editing. IT supervised the drafting and structuring of the manuscript and revised it critically for important intellectual content. All authors read and approved the final manuscript.
